# Pulmonary Tuberculosis

**Published:** 1873-07

**Authors:** A. W. Griggs

**Affiliations:** Professor of the Principles anti Practice of Medicine in the Atlanta Medical College


### PULMONARY TUBERCULOSIS.

BY A. W. GRIGGS, M.D.,

*Professor of the Principles and Practice of Medicine in the Atlanta Medical College.*

Opinions long entertained, and generally believed to be correct, by members of the medical profession, require to be thoroughly analyzed every now and then, the more especially when the doctrines which they inculcate are so important in their relations to the lives of our fellow-creatures. The subject now being considered is one which of late has attracted an unusual attention of pathologists in reference to many points heretofore considered to have been settled; but little progress, compared to the interest of the subject, has been made, until lately, for nearly half a century. Without referring particularly to the various dogmas, in detail, as they have engaged the minds of theorists, I shall only refer to such matters as are deemed worthy of notice in their connection with the origin and development of this fell destroyer of human life. In 1829 I believe that pulmonary tuberculosis was for the first time pronounced to be a constitutional disease. Before that time the symptoms were supposed to depend upon the formation and existence of tubercles in the lungs. The greatest importance was attached to the changes occurring in these organs. We gradually became convinced that tuberculizaticn arose only from a vice of the constitution, and was therefore but the "local expression" of said constitutional disorder. That there is a tuberculous diathesis, which, under exciting causes, induces the tuberculous cachexia, with the concomitant local manifestations, is pretty generally believed. Whether this diathesis, in which the latent and undeveloped elements of tuberculosis are supposed to be present, is capable of hereditary transmission, is answered affirmatively by the great majority. That such hereditary transmission must necessarily occur, we have many facts which go to prove to the contrary. Yet we have heard the doctrine so long, and we have so often seen the children of tuberculous parents affected in the like manner, that we have considered those who escape as exceptional cases, without reasoning out the cause or causes which conduced to their exemption, and we have satisfied our spirit of research with the reflection that the disease remained latent, or dormant, if you please, or that the individual, so to speak, derived his constitution from the healthy parent. We deny the propriety of admitting as true a proposition so broad and comprehensive, involving, as this does, most sacred relations and the highest temporal interests of mankind, unless we do so upon testimony other than the common observation of facts and sequences relating in a general way to the matter under investigation, and at most only able to afford cirsumstantial evidence, however strong. If we could be satisfied with such ordinary court-room testimony, the grave responsibility would still rest upon us to determine the essential nature of the morbific taint, trace it to its prime origin, note the conditions of invasion, and learn the therapeutic means best suited for its removal.

It is well agreed that the *predisposition* may be acquired. When acquired, is it equally likely to become hereditary? It appears that it should be, particularly if it be so, that tubercular corpuscles, germs, or cells, circulate with the blood, contaminate the tissues, and lead to the development of heterologous formations by what is called deposition. Further, it is said that there are some remarkable cases where the diathesis is congenital, although not inherited. Prof. Austin Flint, in his exceedingly philosophic and highly esteemed work on the Practice of Medicine, gives an interesting example of the kind, furnished by a medical friend. Neither parent was tuberculous, nor were their progenitors. They were in good health, the husband being aged fifty-seven and the wife fifty-two. They lost five children of tuberculosis between 1853 and 1861, aged, respectively, at the time of their deaths, 23, 25, 24, and 23 years. It would be interesting to know more about these cases; for instance, the respective temperaments of the parents, and the social position of the family, together with their management (at home) of the children—the hygienic regulations (if any) enforced—the kind of food, and sleeping apartments, etc., etc. I am acquainted with cases similar to the above, but will, however, introduce an instance of exemption in an individual who was born of tuberculous parents, who lost all their other children, several in number, and likewise two grandchildren, of this affection. The little orphan was the only relic of the immediate line, and was quite an infant when his mother died.

He was taken to the home of his lately bereft brother-in-law, who was an educated medical gentleman of the olden time. Suffice it to say that the boy was brought up under the best hygienic regimen; he was fed at a table laden with substantial and luxurious food; he rambled with the little negroes over the fields and forests, developed into almost perfect manhood, without apparent defect or blemish, married soon after he attained his majority, became the father of a healthy progeny, and was accidentally killed when in the enjoyment of good health, in the meridian of life. These examples exhibit some of the freaks of this disease, and may best be accounted for by an examination into the effects of observing or violating the laws of health, etc.

Professor Flint teaches that tubercle is an *exudation*, independent of inflammation, and is non-organizable; that it is but the local expression of a *constitutional condition*, on the account of which the deposit occurs. Further, that the relation which tuberculization bears to the *cachexia*, rather than to the structures in which it takes place, is established by the generally uniform characters presented by the deposit, or substance, in whatsoever organ it may be found. Authors of the present day, generally, are strenuous in the belief that tuberculization is only the declaration of a constitutional disease mostly hereditary. The old doctrine of the lamented Samuel Henry Dickson and others, that this is due to a "*taint*" which is transmitted, unimpaired in virulency, from generation to generation, is believed by many, and made plausibly to appear from numerous statistics. I can not consent to the orthodoxy of such views, nevertheless, they are still held to be correct by many practitioners. We are certainly largely indebted to our predecessors for their honest and laborious researches, which have so greatly lightened our duties by presenting to us so much scientific and well-arranged material, which can be beneficially applied in the construction of other, and it may be equally philosophic, theories. We confess, however, that our opinions are rather those of one who is seeking the solution of the difficult problem, than of him who has completed the analysis and is settled down in his belief. Most authors consent that in the varied forms of tuberculosis there is a perverted and depraved action of the nutritive function, or that there is mal-assimilation, and that this is to be ascribed to the scrofulous *cachexia;* but as to the exact nature of *this*, and as to the conditions necessary in originating it, there is only speculation giving rise to conflicting arguments and beliefs, and various empirical plans of treatment. This will continue to be so until our knowledge shall become more definite—until we can understand more precisely the tissue changes which are constantly going on, molecule by molecule. When physiology shall attain the full dimensions of the great science it is destined to become, and be able to expound the typical force and its relation to nutrition, the pathologist may be able to see his way through the labyrinthian hall, discover the aberrations of nature, and submit his reckonings to the therapeutist, who will perform his part in restoring to supremacy the infracted or subverted law. In this disease therapeutics has gone ahead of its usual guides—has reflected the light of experimental truth and dictated to physiology and pathology more carefully to consider the functions of relation which control the mutual dependency subsisting between the tissue elements and the blood. I am satisfied that there must be a defective vitality or want of typical force in the minute structures of a part, before tuberculization can take place. In some way the equilibrium between the organs of supply and those of removal is lost, and the unrenewed elements begin retrograde action, changing into forms differing from the original structures. These forms are but the debris of the tissues in which they are found, together with the imperfect products of ill-pronounced or abortive inflammation, and increase merely by the disorganizing process under such circumstances peculiar, and by the disintegration of the proximal tissue. Tubercle is, therefore, not deposited. But what shall we say about diathesis? How does the predisposition arise? Or, how is it engendered, and how does it become hereditary? It will be acknowledged by all that these are important questions, requiring patient thought and close investigation for their solution. There must be a point of departure from health.

This may arise from any cause which is capable of exerting continuously for a length of time a morbid depression in, or defective direction of, the assimilative process. The neglect of the plain laws of hygiene; the too free use of highly stimulating and indigestible food, or confinement to that which is poor in quality, or insufficient in quantity, or improperly prepared; excess in the unwise and truly uncomfortable fashions of dress; the miserably murderous eight and ten hour systems of the common schools and manufacturing institutions of the country; that vile and prevalent habit of self-abuse, whereby the nervous system is ruined, and the reproductive organs weakened or dwarfed, variously combined with each other, or kindred violations in the habits of the undeveloped individual; will restrain the great laws of our being, and undermine the very basis of mortal existence.

> "Nature, like liberty, is but restrained
> By the same laws which first herself ordained."

I have enumerated some of the conditions necessary to the origin of the diathesis in a constitution that has otherwise been a model of perfection, as nearly as can be in our tenements. In addition, let me say that nothing is more injurious, or, associated with other irregularities, tends more certainly to engender this diathesis, than poorly ventilated sleeping-rooms, or the crowding of too many in the same beds or apartments. But how does the diathesis become hereditary? Simply by the conferring of similar organic forms and features; or, it may be more directly due to the defective maturation of the ovum of the female, or to a weakened vitality in the sperm-cell of the male—or to both.

It is evident from the views set forth in this paper, if they are correct, that tuberculosis of the lungs may be prevented in a great many instances, and probably arrested in some, by timely interference on the part of the physician.

---

Dr. Caffe, the *doyen* of French medical journalists, in discussing the organic affection in the case of Napoleon III, which, he believes, was "in itself inevitably fatal," remarks that grave organic maladies alternate with constitutional hereditary disorders. Queen Hortense died of cancer in the womb, Napoleon I of cancer in the stomach, and Charles Bonaparte also of cancer.

